# The Remission Phase in Type 1 Diabetes: Role of Hyperglycemia Rectification in Immune Modulation

**DOI:** 10.3389/fendo.2019.00824

**Published:** 2019-12-03

**Authors:** Rong Tang, Ting Zhong, Chao Wu, Zhiguang Zhou, Xia Li

**Affiliations:** ^1^Department of Metabolism and Endocrinology, The Second Xiangya Hospital, Central South University, Changsha, China; ^2^Key Laboratory of Diabetes Immunology, Ministry of Education, National Clinical Research Center for Metabolic Disease, Central South University, Changsha, China

**Keywords:** type 1 diabetes, remission phase, the honeymoon period, immune modulation, hyperglycemia

## Abstract

The remission phase (or honeymoon period) is a spontaneous “temporary cure stage” in type 1 diabetes course, which provides a good human model for studying β-cell protection. The exact mechanisms are still uncertain, but one of the generally recognized mechanisms is that correction of “glucotoxicity” by exogenous insulin therapy leads to “β-cell rest” and β-cell recovery. Beyond this, the remission phase is accompanied by changes in various immune cells and immune molecules, indicating downregulation of immune response, and induction of immune tolerance. The role of hyperglycemia rectification in the regulation of immune response should be emphasized because glucose metabolism is critical to maintain the normal function of immune system. Here, recent evidence of immune modulation based on the rectification of hyperglycemia from multiple aspects such as immune cells, inflammatory cytokines, biomolecules, and cell antigenicity was reviewed. It should be noteworthy that the interaction between glucose metabolism and immune plays an important role in the pathogenesis of the remission phase. The best intervention strategy may be the combination of strict glycemic control and immune modulation to protect β-cell function as early as possible.

## Introduction

Type 1 diabetes mellitus (T1DM) is an autoimmune T-cell-mediated damage to pancreatic β cells, leading to an absolute deficiency in endogenous insulin. Because of the irreversibility of β-cell function decay, continuous exogenous insulin treatment is essential (1). To restore the patient's β-cell function and become insulin independent is the highest goal that scholars have been pursuing for decades. Actually, the primary endpoint of T1DM intervention study is β-cell function protection. Many attempts and strategies have been tried; some have made major progress in immunotherapy, but curing T1DM is still a great challenge to be overcome (2).

During the disease process, some patients have a very special period with tempory recovery of β-cell function, which is called remission phase or “honeymoon period” (3). The concept of “remission phase” was first described by Jackson et al. (4). They observed a rapid decline in demand for exogenous insulin in diabetic children after regular insulin treatment, and hypoglycemic shock was a frequent occurrence during this period. In 1944, Brush (5) gave a more specific description of this phenomenon. Animal experiments also proved that hyperglycemia is a major factor to promote the development of diabetes, and early correction of hyperglycemia is conducive to the recovery of islet function (6, 7). Several subsequent studies confirmed that insulin treatment leads to preservation of residual β-cell function (8, 9).

The remission phase is a short “clinical cure stage,” which provides a decent human model for studying β-cell protection and immune modulation. In-depth studies of the mechanisms of this period may lay a foundation for inhibiting autoimmune response and even achieving clinical cures for T1DM. The fundamental pathophysiological change of the remission phase is a transient recovery of β-cell function (10). The exact mechanisms are still uncertain, but one of the generally recognized mechanisms is that correction of “glucotoxicity” by exogenous insulin therapy leads to β-cell rest. Recent evidence suggested that hyperglycemia rectification might protect β-cell function through other pathways, such as inducing immune modulation (11, 12). The latest study found that the remission phase was accompanied by changes in the frequency of regulatory T cells (T reg), CD25^+^CD127^hi^ cells, memory T cells, Th1 cells, B-cell subsets, neutrophils, and other immune cells (13). Besides the immune cells, a variety of cellular inflammatory factors, immune related molecules may also be related to the remission phase. This review will discuss the possible mechanisms of immune modulation of the remission phase from the aspect of cellular, inflammatory, and molecular levels and focus on the glyco-metabolism in immune cells.

## Immune Cells

### T Cell Subsets

The frequency of some T-cell subsets is closely related to the remission phase, and the correlation might be partly attributable to good glycemic control. Moya et al. (14) found that the high frequency of activated Treg cells (aTreg), CD45RO^+^ memory T cells, and CD25^+^CD127^hi^ cells in T1DM patients had a positive correlation with the duration of remission phase, and these correlations only existed in the condition of satisfactory glycemic control. A study in 2018 also found that the remission phase was accompanied by dynamic changes in Treg cells, Th1 and Tc1-cell subsets (13), with the decreased number of Treg cells and increased Th1 and Tc1 cells at the end of the remission phase. A recent study that examined a variety of immune cell subsets and cellular molecules in newly diagnosed children with T1DM found that the level of memory-regulatory T cells (mTreg) decreased after the appearance of clinical symptoms and aTreg and Th17 cells were significantly elevated in the first year of onset (the remission phase often occur) (15). The mTregs were also noted to be positively correlated with C-peptide in T1DM patients (16). Evidence of the obvious dynamic changes in Tregs in the different stages of remission phase suggested that the immune response during the remission phase was most likely related to the frequency and function of Tregs.

Treg cells (CD4^+^CD25^+^T cells) play a critical role in the induction and maintenance of peripheral tolerance (17–19). Dysfunction of Treg cells has been found to be one of the key pathogenesis of autoimmune diabetes (20, 21). There is one study that showed that presence of Tregs to Teffs dominance is the underlying cause of the remission phase (22). Considering the crucial roles of glucose metabolism in T-cell function, it has been expected that hyperglycemia rectification may be a trigger event for dominance of Tregs in the remission phase.

As early as in 2008, Maciver et al. (23) found that the energy that is supplied by glucose is necessary for T cells to sustain growth, proliferation, and immune activity. T cells cannot produce the appropriate cytokines, such as IFN-γ, to exert an immune effect without adequate glucose uptake (23). This suggests that the immune function of T cells requires a normal state of glucose metabolism. Treg cells have also been implicated in glucose metabolism in numerous studies (24, 25). Recent study showed that the growth and differentiation processes of Tregs and Th17 are significantly dependent on glycolysis and aerobic phosphorylation (26). In this regard, although there is no direct evidence available, it is rational to deduce that rapid rectification of hyperglycemia at the initial stage of T1DM might help to recover the impaired function of Treg cells and downregulate the immune response that is characterized by remission phase. Indeed, in the study of Moya R, the frequent changes of aTreg, CD25^+^CD127^hi^ cells, and CD45RO^+^ memory cells were correlated with better blood glucose control (14), which may serve as indirect evidence for this hypothesis.

In addition to Tregs, other T-cell subsets are less reported in the remission phase. In the previously mentioned study, Moya showed that the CD25^+^CD127^hi^-cell frequency was significantly correlated with that of aTregs. In this way, it could be speculated that good glycemic control might also restore the function of CD25^+^CD127^hi^ cells, which promote the development of Tr1 cells through CD44 and CD44v6 signaling pathways (14). Tr1 and memory T cells have also been demonstrated to inhibit autoreactive inflammation (27, 28), but their roles in the remission phase have not been reported so far.

### B-Cell Subsets

Recent studies have shown that B lymphocytes also play an indispensable role in the pathogenesis of the autoimmune diabetes (29, 30). A study showed that the frequency changes of marginal zone B cells (MZB), follicular B cells (FoB), and other B-cell subsets were associated with T1DM, and the regulatory B cells (Breg) have immunomodulatory effects (30). Limited reports showed that the remission phase was accompanied by changes in B-cell frequency (13, 15). Glucose metabolism had also been shown to play an important role in the normal functioning of B cells. Therefore, it may be hypothesized that hyperglycemia rectification during the remission phase affects the frequency of B cells.

Studies on the relationship between B-cell subsets and the remission phase are rare. In 2018, Fitas et al. (13) found that the absolute number and relative frequency of B cells decreased significantly during the disease course when compared with the onset stage and hit the lowest level in the remission phase. A recent study found that the Breg cells significantly increased during the remission phase (15). In addition, the study showed that the intervention of the anti-CD20 monoclonal antibody rituximab can extend the duration of the remission phase to 2 years (31). These combined results suggested that B lymphocytes are involved in the pathogenesis of the remission phase.

Among all B-cell subsets, the Breg subpopulation with immunomodulatory effects is a research hotspot. An animal studied showed that Breg subpopulations that secrete IL-10 (CD1d^hi^CD5^+^, B10) can effectively inhibit the proliferation of effector T cells and alleviate the decline in β-cell function by secret IL-10 (32). Breg cells were increased in the early recovery stage of T1DM (15), and the B10 subgroup was positively correlated with fasting C-peptide level and negatively correlated with HbA1c of T1DM patients (30). The evidence suggests that B-cell subsets participate in the process of the remission phase. In addition, it was reported that B10 cells can reduce T-cell-mediated islet transplant rejection by promoting Treg-cell development and inhibiting pro-inflammatory Th1-cell activity (33). Considering this situation, it is a high value to investigate the effects and mechanisms possible of B10-cell subsets in the remission phase.

At present, studies about glucose metabolism and immune cells are mainly concentrated in T cells and macrophages. Studies of the relationship between B cells and glucose metabolism are rare. An *in vitro* study showed that B-lymphocyte activating factor, lipopolysaccharide, and B-cell receptor rapidly accelerate glucose uptake and glycolysis, which provide rapid energy for cell proliferation (34). In addition, Kojima et al. (35) reported that hypoxia-inducible factors play a part in upregulating glycolysis during B-cell development. Furthermore, hypoxia-inducible factor-1α plays a critical role in the expansion of B10 cells and the expression of IL-10 (36). It is suggested that the regulation of B-cell energy metabolism is essential for its development and function. Therefore, the rectification of hyperglycemia may affect the normal function of B-cell subsets and participate in the remission phase immune modulation process. However, there is no relevant research evidence yet.

### Other Immune Cells

Besides T and B cells, other immune cells such as natural killer (NK) cells, monocytes, and neutrophils may also participate in the remission phase of T1DM. Fitas et al. (13) reported that the number of neutrophil and NK cells was significantly reduced at the onset of T1DM and began to recover during the remission period, and low percentage of NK cells and high percentage of neutrophils were positively correlated with the duration of the remission phase. The number of mononuclear cells also decreased after the onset of T1DM and reached the nadir in the first year (15). These changes of the immune cells in peripheral blood suggest an active extravasation to target tissues, probably contributing to the downregulation of the autoimmune response (16).

## Cytokines

The roles of pro-inflammatory and anti-inflammatory cytokines in the pathogenesis of T1DM have been known for a long time. Numerous cytokines have been tested as possible biomarkers for the remission phase. Hvidoere's team found that Th1-related chemokines CCL5 decreased in patients with remission and was positively correlated with HbA1c, while CCL3 increased in these patients but negatively correlated with C-peptide (37). Furthermore, patients during the remission phase were accompanied with decreased IFN-γ and TGF-β levels, and high levels of IL-10 were in parallel with good glycemic control (15, 38, 39). However, some inconsistent results had been reported, and pro-inflammatory IL-6 was shown to be elevated in the remission phase while demonstrated a positive correlation with glycemic control (40). When different cytokines were taken together and defined patients according to multiple cytokines, that is, low-responder patients who did not detect any anti-inflammatory (IL-4, IL-10, and IL-13) and pro-inflammatory factors (TNF-α) and high-responder patients with at least one cytokine detected, it was found that low responders had higher C-peptide levels and longer remission duration than high responders (13), suggesting the downregulation of pro-inflammation in the remission phase.

Glucose metabolism is implicated in the change pattern of cytokines. Soluble interleukin-7 (IL-7) receptor α (sCD127) expressed on the surface of T cells, when combined with IL-7, has an antagonistic effect on IL-7 signaling pathway and IL-7-mediated T-cell proliferation (41). It is worth noting that hyperglycemia resulted in a glycosylated form of sCD127 that was ineffective as an IL-7 antagonist and glycosylated sCD127 was found in patients with T1DM (42), which provided new evidence that hyperglycemia regulates the immune network by acting on immune molecules.

## Immune Molecules

The role of inhibitory immune-related molecules such as CTLA4 is crucial in the pathogenesis of T1DM. Recently, programmed cell death-1 (PD-1), a transmembrane glycoprotein belonging to CD28/CTLA4 family, which is acting as an important immunosuppressive molecule that is mainly expressed on activated T or B cells, is regaining the attention in the field of T1DM as well as in oncology (43). It was shown that the infiltration of autoimmune islet inflammation and islet antigen reactive T cells was aggravated when PD-1 or PD-L1 was blocked (44). PD-1/PD-L1 as a therapeutic target can effectively inhibit autoimmune T-cell response in non-obese diabetic mice and reverse diabetes (23). The widespread application of PD-1/PD-L1 antibody therapy is an epoch-making event in the field of oncology, but their side effects as PD-1 inhibitor induced T1DM have prompted us to explore the roles of PD-1/PD-L1 in T1DM and its possible participation in the remission phase. It is noteworthy that the expression of PD-L1 on β cells could alleviate autoimmune attacks and resist T-cell-mediated destruction (45, 46), making PD-L1 as a potential biomarker for recognizing β cells that resist the immune attack. In this sense, it is of particular interest to explore whether PD-1/PD-L1 expression of immune cells and β cells could be a predictor for the occurrence and duration of the remission phase.

## Immunogenicity of Islet β Cells

A study showed that some β cells can survive a long time without being immune attacked by reducing the expression of diabetes-associated antigens IGRP, Chga, Gad1, and Ins1/Ins2 (46). This suggests that changes of β-cell antigenicity play an important role in the immune destruction process. The effect of blood glucose on the immunogenicity of β cells has long been documented, and a hyperglycemic environment could stimulate antigen expression (47–49). A new study found that resident macrophages in islets could take up the β endocellular insulin-containing granules through its filopodial and present insulin peptides to insulin-reactive T cells and the antigen presentation was increased after high-glucose stimulation in non-obese diabetic mice (50). Skowera et al. (51) showed that the expression of cell-specific proinsulinogen (PPI) signaling epitopes increased in long-term hyperglycemic concentrations, leading to increased PPI-specific CD8+ T-cell killing of cells and accelerated β-cell death. Accordingly, a significant decrease of autoantigen expression of β cells was observed after insulin treatment (52, 53). Taken together, it was deemed that correction of the “glucotoxicity” during the remission phase may downregulate immune response by reducing the immunogenicity of β cells.

## Discussion

To sum up, patients in remission phase were accompanied by changes in the frequency of various cells and immune molecules, including increased aTreg, Th17, Breg, and neutrophil cells to varying degrees, decreased B cells, NK cells, IFN-γ, and lower β cell immunogenicity, partly due to hyperglycemia rectification. Meanwhile, duration of the remission phase is also related to a variety of cells and molecules, such as aTreg, CD25^+^CD127^hi^ cells, CD45RO^+^ memory cells, neutrophil, and IL-10, which are positively correlated, and NK cells and some inflammatory molecules (IL-4, IL-10, IL-13, and TNF-γ), which are negatively correlated (Table 1). The occurrence of remission phase is based on the immune modulation as well as β-cell rest, and rectification of hyperglycemia is likely to be the main cause of this immune change. Although the exact mechanisms are not clear, it could be deemed that controlling hyperglycemia not only is important to β-cell rest but also has vital effects on various immune components. The best way to intervene in T1DM may be to combine strict glycemic control with immune intervention to protect β-cell functions as early as possible and as long as possible.

**Table 1:** The immunological changes during the different stage of the remission phase.

**Immunological markers**	**Changes during the remission phase**	**References**
aTreg, Th17, Breg, Neutrophil, CCL3, IL-6	Increased at the beginning of the remission phase	(13, 15, 37, 40)
B cells, mononuclear, NK cells, CCL5, INF-γ, TGF-β, β-cell immunogenicity,	Decreased at the beginning of the remission phase	(13, 15, 37, 38, 52)
aTreg; CD4^+^CD25^+^CD127^hi^ cells; CD4^+^CD45RO^+^ memory cells; Neutrophil; IL-10,	Positively correlation with the length of the remission phase	(13, 14, 39)
NK cells; IL-4; IL-13; TNF-α,	Negatively correlation with the length of the remission phase	(13)

Although there are several published studies on the remission phase of T1DM (54, 55), reviews focusing on mechanisms of immune modulation by hyperglycemia rectification are lacking. Our review has some strength in providing a comprehensive overview and new evidence on the interaction between glucose metabolism and immune regulation from multiple aspects such as immune cells, inflammatory cytokines, biomolecules, and cell antigenicity. The importance of immune-glycometabolism should be emphasized in the pathogenesis of the remission phase.

## Author Contributions

RT wrote the manuscript. TZ contributed to the Cytokines and Immune molecules section. CW searched part of the references. XL provided ideas. ZZ polished the article.

### Conflict of Interest

The authors declare that the research was conducted in the absence of any commercial or financial relationships that could be construed as a potential conflict of interest.
